# A Deep Learning-Based Workflow for Dendritic Spine Segmentation

**DOI:** 10.3389/fnana.2022.817903

**Published:** 2022-03-17

**Authors:** Isabel Vidaurre-Gallart, Isabel Fernaud-Espinosa, Nicusor Cosmin-Toader, Lidia Talavera-Martínez, Miguel Martin-Abadal, Ruth Benavides-Piccione, Yolanda Gonzalez-Cid, Luis Pastor, Javier DeFelipe, Marcos García-Lorenzo

**Affiliations:** ^1^VG-LAB, Universidad Rey Juan Carlos, Móstoles, Spain; ^2^Instituto Cajal, Consejo Superior de Investigaciones Científicas (CSIC), Madrid, Spain; ^3^Laboratorio Cajal de Circuitos Corticales, Centro de Tecnología Biomédica, Universidad Politécnica de Madrid, Madrid, Spain; ^4^Departament de Matemàtiques i Informàtica, Universitat de les Illes Balears, Palma, Spain; ^5^E-Health and Multidisciplinary Telemedicine Through Cyber-Physical Intelligent Systems, IdISBa, Palma, Spain; ^6^Research Center for Computational Simulation (CCS), Madrid, Spain

**Keywords:** automatic 3D image segmentation, artificial neural network, confocal microscopy, reconstruction algorithms, pyramidal cells

## Abstract

The morphological analysis of dendritic spines is an important challenge for the neuroscientific community. Most state-of-the-art techniques rely on user-supervised algorithms to segment the spine surface, especially those designed for light microscopy images. Therefore, processing large dendritic branches is costly and time-consuming. Although deep learning (DL) models have become one of the most commonly used tools in image segmentation, they have not yet been successfully applied to this problem. In this article, we study the feasibility of using DL models to automatize spine segmentation from confocal microscopy images. Supervised learning is the most frequently used method for training DL models. This approach requires large data sets of high-quality segmented images (ground truth). As mentioned above, the segmentation of microscopy images is time-consuming and, therefore, in most cases, neuroanatomists only reconstruct relevant branches of the stack. Additionally, some parts of the dendritic shaft and spines are not segmented due to dyeing problems. In the context of this research, we tested the most successful architectures in the DL biomedical segmentation field. To build the ground truth, we used a large and high-quality data set, according to standards in the field. Nevertheless, this data set is not sufficient to train convolutional neural networks for accurate reconstructions. Therefore, we implemented an automatic preprocessing step and several training strategies to deal with the problems mentioned above. As shown by our results, our system produces a high-quality segmentation in most cases. Finally, we integrated several postprocessing user-supervised algorithms in a graphical user interface application to correct any possible artifacts.

## 1. Introduction

Pyramidal cells are the most abundant neurons in the cerebral cortex. These cells are the principal source of intrinsic excitatory cortical synapses, and they represent the major cell type projecting to other cortical and subcortical regions. Furthermore, they form the vast majority of cortical connections, and their dendritic spines (for simplicity, spines) are the main postsynaptic targets of excitatory synapses (DeFelipe and Fariñas, 1992). Spines were discovered by Cajal in 1888 (DeFelipe, 2015) and, since then, thousands of studies have been conducted, as these structures are critical to learning, memory, and cognition (Harris and Kater, 1994; Bourne and Harris, 2007; Spruston, 2008; Yuste, 2010; Kandel et al., 2014). Alterations of spines have been related to several neurodegenerative diseases and pathologies, including Alzheimer's disease (Fiala et al., 2002; Yu and Lu, 2012; Merino-Serrais et al., 2013).

Spine structure is considered crucial for neural signal transmission and changes in its morphology are related to synaptic plasticity (Segal, 2005; DeFelipe, 2006; Yuste, 2010). Over the years, much effort has gone into the study of spine density, shape, size, and distribution at the structural level. Nevertheless, the segmentation techniques used and accepted by the community still rely on an expert to supervise the process. The large number of these structures makes the user-guided extraction process tedious and time-consuming, reducing the segmented data available for analysis, making the development of automatic techniques necessary (Zhang et al., 2010; Mukai et al., 2011). In recent years, light microscopy techniques, such as confocal microscopy or two-photon microscopy, are preferred over electron microscopy for two main reasons: (i) they allow larger regions to be captured (Arellano et al., 2007; de Lagran et al., 2012) and (ii) some of them can be used in live brain tissue and allow the study of its structural changes over time (Hoover and Squier, 2013). The main constraint of light microscopy is the limited optical resolution of the images. The relatively low quality of these images, compared to electron microscopy, hinders the segmentation process, leading to difficulties in the identification of true spine boundaries (Mishchenko et al., 2010; Mukai et al., 2011). For example, it is relatively common for the necks of the spines of pyramidal cells to be so thin that they are close to the optical resolution limit of the imaging techniques, since their small size limits the quantity of fluorophore that can be absorbed (Son et al., 2011).

Deep learning (DL) techniques are currently a standard in automatic segmentation. They have been successfully applied to several problems in the biomedical field (Minaee et al., 2021). Generally, and particularly in this field, the most widely used deep artificial neural networks belong to the supervised learning subcategory. These techniques train deep artificial neural networks using examples. The examples consist of pairs of the inputs and the result (desired outputs) of the task to be performed. The set of examples is known as ground truth (GT), and is split into two: the training set used to train and validate the network and the test set used to evaluate the network performance once trained. During the training phase, the network receives the training set inputs and computes outputs. An error function (loss function) compares the network outputs with the GT outputs (correct results), and the model^1^ parameters are fitted to reduce the error. Part of the training set is reserved for validation during training. This data set allows the adjustment of the network architecture. Regarding our image segmentation problem, the inputs are the confocal microscopy images, whereas the GT results and the network outputs are segmented images^2^ called labeled images. In the present work, since we are using intracellularly labeled neurons from confocal microscopy, we will refer to the labeled images as annotated, reconstructed, or segmented images to avoid any possible confusion. After the training phase, the goal is to predict the correct outputs for new inputs. The test set provides previously unseen examples for unbiased estimation of the model's performance.

The success of the training process depends, to a large extent, on the size and quality of the GT. The GT is often built from expert knowledge. Scarce and weak annotated data sets are common problems in biomedical imaging (Tajbakhsh et al., 2020). In the context of spine segmentation, these issues are, especially relevant. Current spine segmentation techniques require user intervention, making the process costly and time-consuming. Furthermore, even for data sets where a high number of spines are reconstructed, there are some issues inherent to confocal microscopy (e.g., some spine neck diameters are below the optical resolution limit) or related to the way spines are reconstructed (e.g., to capture the complete morphology of spines, sometimes it is necessary to use several surfaces with different intensity thresholds, and sometimes there are gaps in between—see Section 3). Such issues need to be tackled. In this research, we used a data set composed of 8,000 spines extracted from high-quality confocal images, and most of them were segmented accurately. Even with a data set of these characteristics, we had to implement several techniques to train DL models and address typical problems in this field, such as overfitting.^3^ For this reason, despite their popularity, these types of techniques have not yet been applied to this problem (Smirnov et al., 2018).

In this article, we evaluate the feasibility of using artificial neural networks for spine segmentation. For this purpose, we built a GT of spine and dendritic shaft reconstructions from confocal microscopy images. This data set was generated using user-supervised algorithms. We propose an automatic data preprocessing technique to generate a GT with sufficient quality to train a reliable artificial neural network. Subsequently, we used the GT to train and evaluate several variations of the two main state-of-the-art DL architectures. The training phase was adapted to solve some of the remaining problems of the GT. Our final model shows promising results and some limitations. We developed a graphical user interface (GUI) application to allow experts to correct the automatic segmentation when needed and overcome these limitations. Our GUI application stores the user-guided corrections to improve the quality of the training data set and to train more accurate models in the future. We believe that our work opens the door to the use of DL for automating the segmentation of spines.

## 2. Related Studies

Commercial tools, currently endorsed by the community, such as Imaris (Bitplane AG, Zurich, Switzerland) or Neurolucida (MicroBrightfield, VT, USA), are heading toward automation of the segmentation process. Nonetheless, they still rely on user-supervised techniques. Traditionally, Imaris allowed the reconstruction of spines using algorithms based on user-selected thresholds. Imaris Filament Tracer represents a significant step toward automating the segmentation and analysis of neural structures at the cost of simplifying the spine geometries. Autospine is Neurolucida's module for spine detection, reconstruction, classification and quantitative measurement. Users must configure a set of parameters for a rather accurate segmentation. Issues such as detached spines are common. Both environments complete their suites with editing tools that enable the correction of segmentation problems. Imaris and Neurolucida were successfully applied in relevant studies in the field (Swanger et al., 2011; De Bartolo et al., 2015; Gao et al., 2019; Henderson et al., 2019).

In recent decades, several studies have addressed the spine segmentation problem. Intensive research on the topic is motivated by the interest of the neuroscientific community in these structures. A recent survey on detection, segmentation, measurement, and classification of spines by light microscopy was presented by Okabe (2020). In this study, the author classified the algorithms depending on the dimensions of the input images. Early techniques approached this problem from a 2D perspective. However, the increase in computational capacity has shifted this focus toward 3D images. Most of the *ad hoc* methods begin by estimating the centerline of the dendritic shaft and spines. The work presented by Koh et al. (2002) proposed one of the first techniques capable of segmenting 3D images. Their technique computes a dendritic skeleton from a binarized image (using a fixed threshold). The spine detection phase was designed to deal with detached spines. Similarly, Son et al. (2011) calculated a binarized image to obtain the dendritic shaft and spine centerlines. They then recomputed the spine borders from automatically detected feature points, using active contour segmentation. Their technique separates overlapping spines using a watershed-based algorithm. The method proposed by Zhang et al. (2010) is also based on the medial axis extraction. However, instead of using a binarized image, the authors obtained the medial axis first and then calculated the seed points to perform a fast-marching algorithm. Mukai et al. (2011) also computed the dendrite boundary from its centerline and later segmented the spines detecting features from the Hessian matrix eigendecomposition of the image intensity function. Whereas most techniques calculate features and seed points automatically or assist the expert in the process, some modern algorithms, such as the one proposed by Basu et al. (2018), still require the user to select them manually. The authors of the same study have recently tested the accuracy of their approach on *in vitro, ex vivo*, and *in vivo* two-photon microscopy images (Das et al., 2021). Although the approaches mentioned above are the most widespread, some authors explore other alternatives. For example, He et al. (2012) used non-linear degeneration equation to enhance the morphological differences between dendritic shafts and spines. After that, spine detection and segmentation can be achieved in 3D by applying global thresholding.

In recent years, machine learning (ML) has become one of the most widespread approaches for image processing, including segmentation. However, only a few ML techniques for spine segmentation can be found in the literature. In the context of ML-based segmentation, Erdil et al. (2015) proposed a support vector machine model to classify spines, and then used this information to segment them by applying active contours. Blumer et al. (2015) presented a spine detection and segmentation algorithm based on a statistical dendrite intensity model and a spine correspondence probability model. The statistical model was trained on synthetic fluorescence images generated from serial block-face scanning electron microscopy, and the method was tested on real two-photon data sets. Smirnov et al. (2018) presented an application to detect spines using ML. First, they binarize the image and extract the backbone to identify the dendrite (in a similar way to most *ad hoc* algorithms). They do not apply machine learning to automatize the whole segmentation process since they consider that current data sets are not large enough to train complex DL networks. They then classify the spines using several parameters calculated from the dendritic contour. Unfortunately, their technique only works on 2D images (maximum intensity projections).

DL models have demonstrated outstanding performance in image segmentation. In this regard, Minaee et al. (2021) reviewed the most relevant neural networks to segment images. Two architectures stand out in the medical image domain: U-Net and V-Net. The former was proposed by Ronneberger et al. (2015). A year later, Çiçek et al. (2016) extended this architecture to deal with 3D images (3D U-Net). That same year, Milletari et al. (2016) presented the V-Net for 3D medical image segmentation. One of their most important contributions was the introduction of a new loss function based on the Dice coefficient to deal with highly imbalanced binary classes.

Despite the success of the above-mentioned architectures, the frequent lack of quality of the annotated image data sets hinders their application. Tajbakhsh et al. (2020) classified the main problems of these data sets into two groups: weak-annotated and scarce data sets. Additionally, they reviewed several solutions proposed by the scientific community. Data augmentation and postprocessing are frequently used to overcome these limitations. Moreover, when these approaches do not solve the problem completely, some authors develop tools to reduce the expert annotation effort (Sakinis et al., 2019; Zheng et al., 2019). Our work follows a similar approach. While exploring the feasibility of DL models for spine segmentation, we have developed a user-supervised tool for editing our neural network predictions. We plan to use the corrected data to design new architectures in future research.

## 3. Materials and Methods

**Figure 1:**
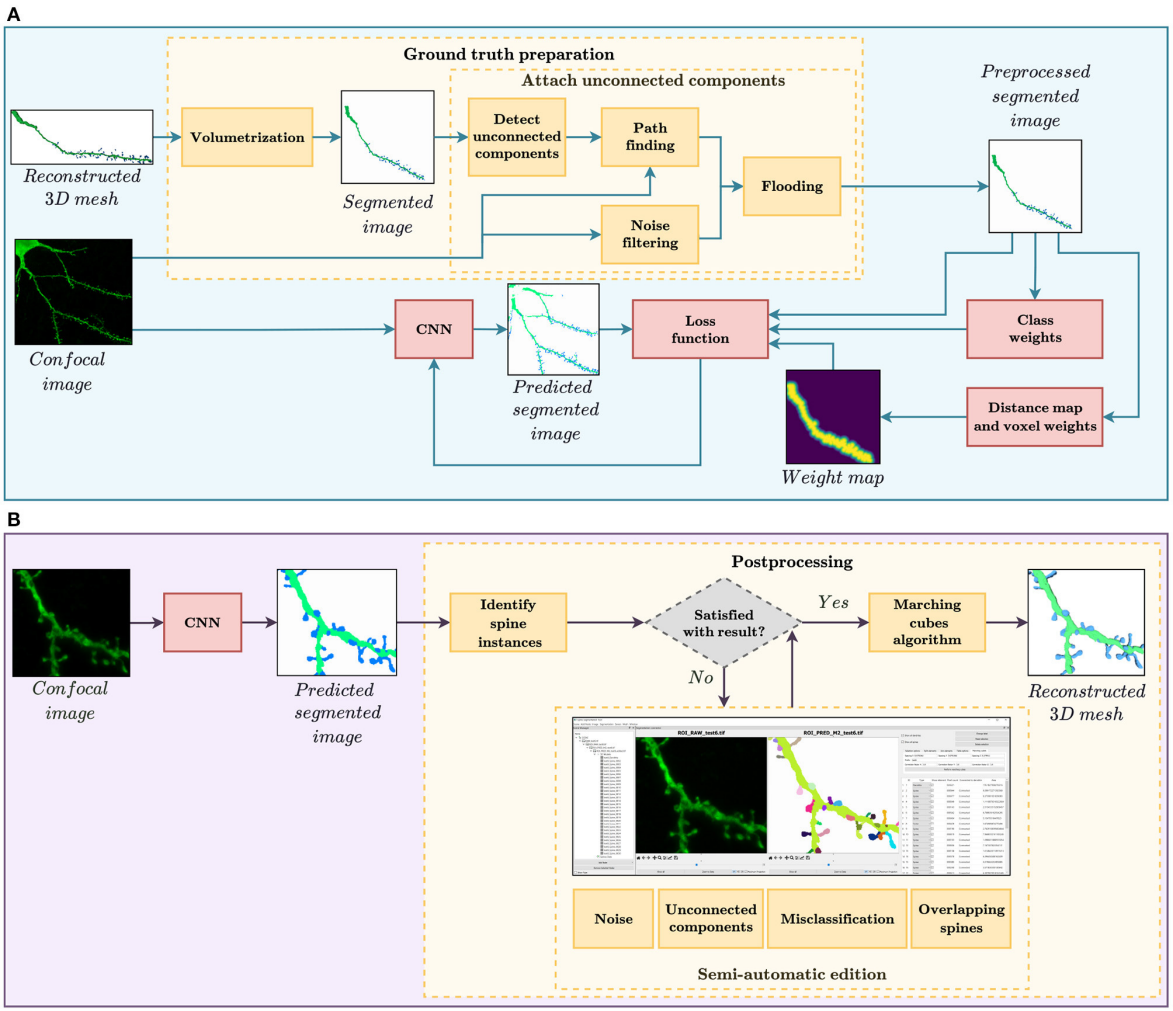
**(A)** GT preparation and training workflow. The GT preparation steps are shown in yellow. First, the reconstructed 3D meshes of dendritic shafts and spines are transformed into a voxel-based segmented image. Then, unconnected parts of the dendritic shaft and its spines are detected and corrected automatically. Among other strategies, the loss function, used to train our convolutional neural networks (CNN), considers several types of class and pixel weights to alleviate training data set problems. **(B)** Automatic segmentation and postprocessing workflow. First, our CNN model provides a fully automatic segmentation. Then, the spine instances are identified. Users can then correct the CNN prediction through our GUI, if necessary. Finally, our system generates the 3D meshes of the segmented dendritic shaft and spines.

In this study, we evaluate the feasibility of using deep learning techniques to automate spine segmentation. For this purpose, we developed the two workflows shown in Figure 1. The pipeline shown at the top of the image illustrates the GT preparation and the model training, whereas Figure 1B displays the procedure of segmenting new data. The software implementation for both pipelines is publicly available for its non-commercial use under an open-access license.

The first step for creating our GT was the data collection phase (Section 3.1). Domain experts manually reconstructed dendritic shafts and spines from confocal images. The most prevalent issues of these segmentations were removed during the preprocessing (Section 3.2) and training phase (Section 3.3) to increase the GT quality. As shown by the results, our best neural networks generated accurate segmentations in most scenarios. Nevertheless, to integrate our models into the neuroanatomists' workflow, we present a tool that allows correction of the networks' outputs and reconstruction of the surface meshes of spines and dendritic shafts (Section 3.4).

### 3.1. Data Acquisition and Preparation

**Figure 2:**
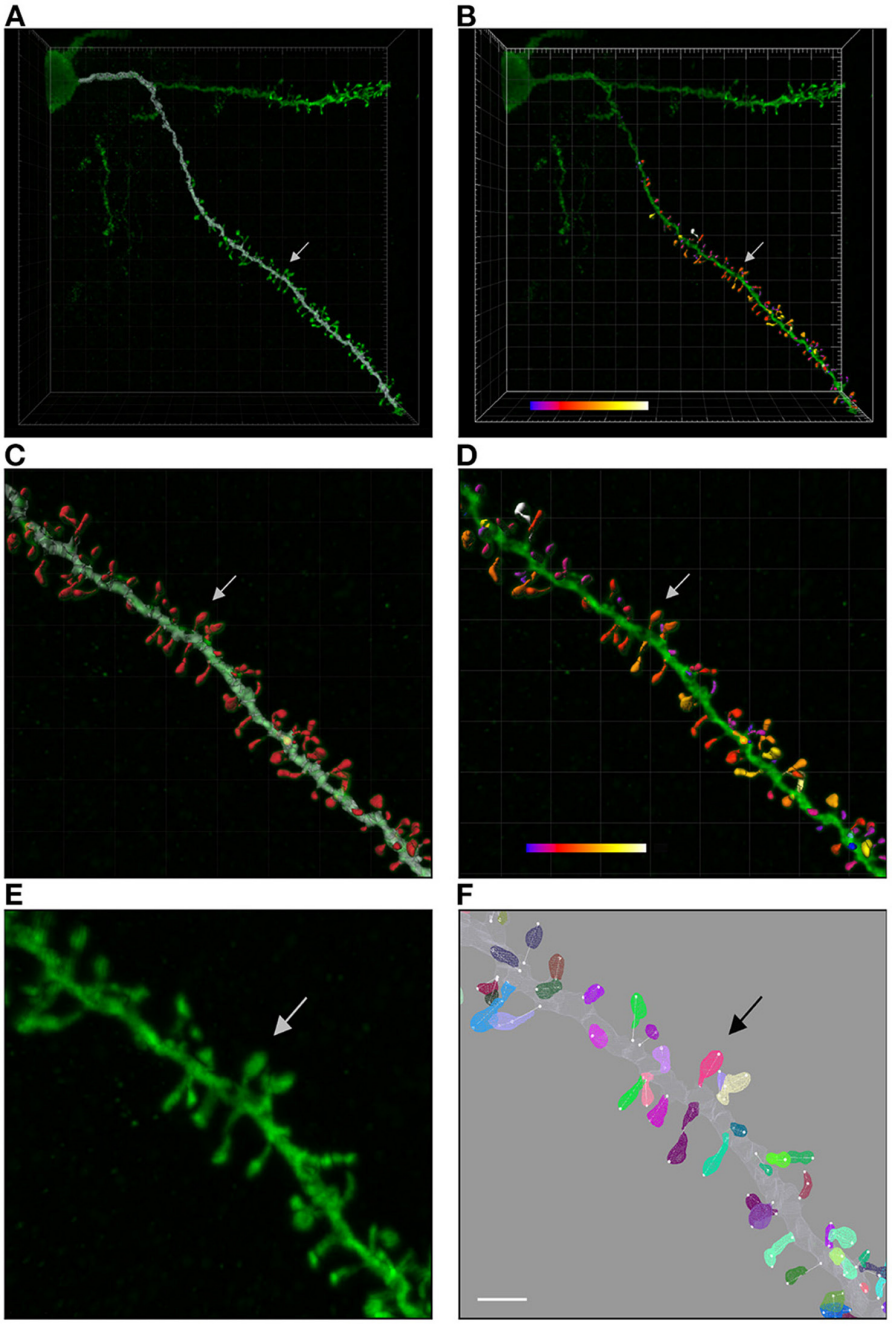
**(A)** Shows a confocal microscopy image of a proximal basal dendritic segment from an intracellularly injected layer III pyramidal neuron of the human cingulate cortex. Dendritic shaft reconstruction is shown in white. **(B)** Shows the three-dimensional reconstruction of each dendritic spine from the dendritic segment colored in white in **(A)**. **(C,D)** Display higher magnification images of **(A,B)**, showing the dendritic shaft and spine reconstructions. An estimation of the spine volume values is shown in **(B,D)** by color codes (blue-white: 0.0–0.899 μm^3^). **(E,F)** Present higher magnification images of the dendritic segment shown in **(C,D)**. Spines are randomly colored in **(F)** to clearly visualize the surface meshes that were manually created and assigned to each spine. Spine lengths (white lines) were also measured in 3D. Arrows indicate the same spine in all panels. Scale bar: 10 μm in **(A,B)**; 4 μm in **(C,D)**; and 2.7 μm in **(E,F)**.

The data set used in the present study was taken from Benavides-Piccione et al. (2013). It includes confocal stacks of images and the corresponding 3D reconstructions of apical and basal dendritic shafts and spines from layer III pyramidal neurons from the cingular cortex of two human males (aged 40 and 85 years old). A total of 8,926 spines were 3-dimensionally reconstructed along 6.35 mm of dendritic length of 16 main apical and 60 basal dendritic segments. As shown in Figure 2, basal spines were reconstructed from the soma to the distal tip of dendrites. The main apical dendritic spines were reconstructed at a distance of 100–200 μm from the soma (since dendrites were virtually devoid of spines for the first 80–90 μm). Apical collateral spines were not included in the analysis. Further information regarding tissue preparation and dendritic labeling is outlined in Benavides-Piccione et al. (2013). Dendritic segments were imaged at high magnification (×63, glycerol) using Leica TCS 4D confocal scanning laser attached to a Leitz DMIRB fluorescence microscope, using an excitation wavelength of 491 nm to visualize Alexa fluor 488. The images were 1,024 voxels wide and high, with a variable depth. The voxel size was 0.0751562 × 0.0751562 × 0.279911μm. For each stack of images, confocal parameters were set such that the fluorescence signal was as bright as possible while ensuring that there were no saturated pixels. Spines were individually reconstructed using Imaris 6.4.0 (Bitplane AG, Zurich, Switzerland). For each spine, a threshold was selected to constitute a solid surface that exactly matched the contour of each spine. However, sometimes it was necessary to use several surfaces of different intensity thresholds to capture the complete morphology of a given spine (Benavides-Piccione et al., 2013).

### 3.2. Ground Truth Preparation

**Figure 3:**
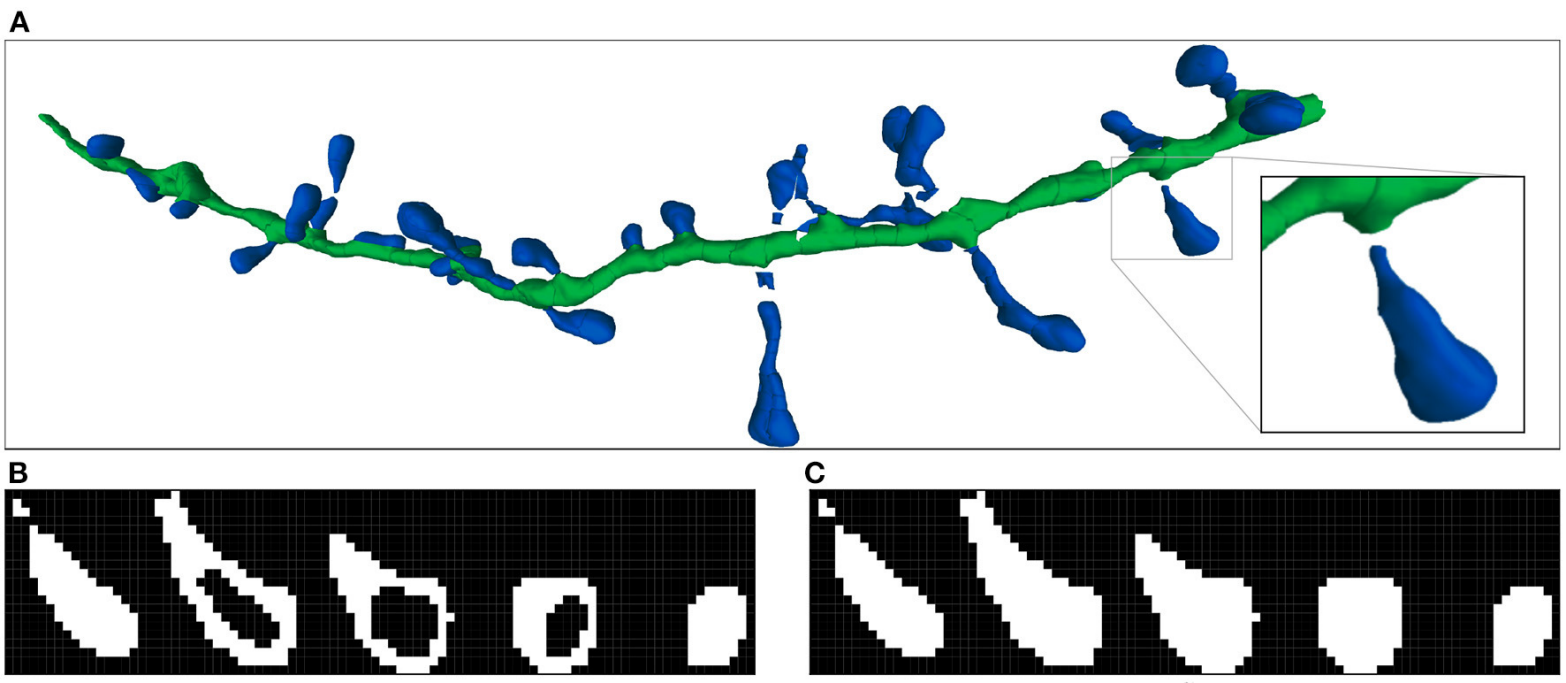
Imaris 3D segmentation and the two steps of the voxelization process: **(A)** Shows an example of the reconstructed 3D meshes of a dendritic shaft (dark green) and spines (dark blue). The processed spine is highlighted. **(B)** Illustrates the first step of volumetrization. The voxels of the image are projected into surface coordinate space as 3D boxes. If the projected box of a voxel contains or collides with a surface polygon, the voxel is marked. It should be noted that the voxel projected boxes and surface polygons are 3D structures. Therefore, the surface can collide with more than one voxel in the same stack plane. **(C)** Shows the second step, where the inner voxels are also marked. All spine stack planes are shown.

Imaris stores the surfaces of the spines and dendritic shaft using polygonal meshes. We transformed the dendritic shafts and spine mesh-based representations (B-reps) into voxel-based segmented images. All the segmented image voxels were classified either as background (0), dendritic shaft (1), or spine (2). The algorithm used is divided into two steps (see Figure 3): (i) it traverses the mesh polygons of spines and dendritic shafts, and classifies the voxels of the segmented image that contain them; (ii) it segments the inner voxels of the spines and dendritic shaft by applying the algorithm described by Suzuki et al. (2003) to detect connected groups of voxels. Our algorithm only works on closed surfaces. Imaris uses user-selected thresholds to compute closed isosurfaces. Nevertheless, other meshes can be processed using a hole-filling algorithm, such as the one proposed by Zhao et al. (2007). Other spine segmentation tools and algorithms, such as the one proposed by Das et al. (2021), provide an annotated image as output instead of surface-based representations. In these cases, the volumetrization step is not necessary to build the GT.

At this point, our data set suffered problems that hindered our DL model training. As stated above, Tajbakhsh et al. (2020) classified the main GT problems into two groups: scarcely and weakly annotated data sets. We implemented a preprocessing step to tackle some of these issues, whereas the rest were dealt with during the training phase (see Section 3.3).

**Scarce annotations:** Difficult problems require complex neural network architectures, and complex architectures require large training data sets. If the GT is not sufficiently large, overfitting is likely to happen. When overfitting occurs, the network learns the training data but is not able to predict unseen images correctly. In our data set, 8,926 spines were distributed along 76 dendritic segments. This data set was not sufficient for training our networks without overfitting. As previously explained, data augmentation is the most commonly used technique to avoid overfitting. We tested the usefulness of this approach by increasing our data set applying isometric transformations to preserve shape and distance. We implemented symmetries, rotations, and translations that keep voxel-to-voxel correspondence between the original and the transformed image. Additionally, we tested other techniques (such as early stopping, dropout, and other regularizations) during the training phase to prevent overfitting.

**Figure 4:**
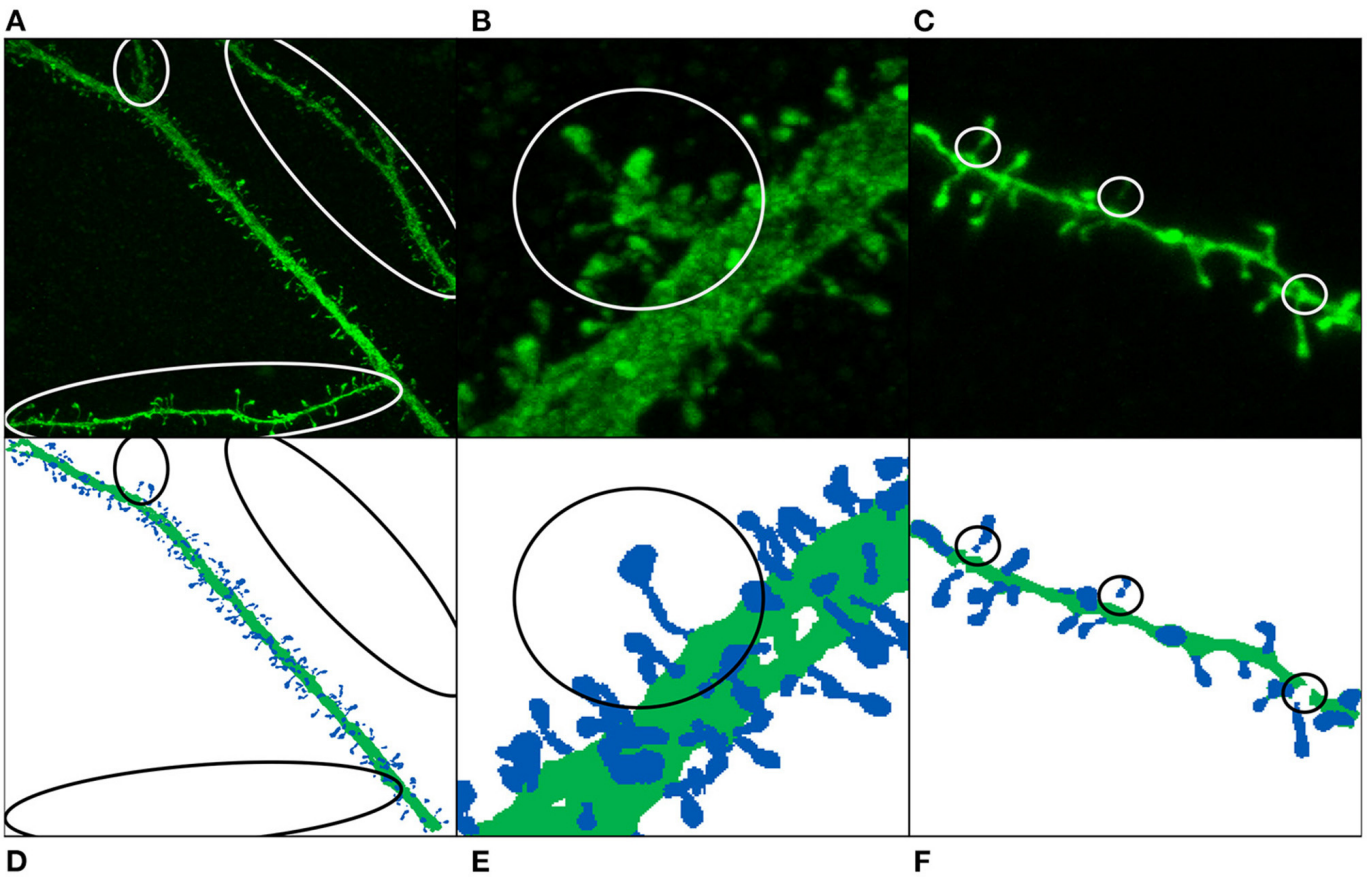
Image annotation issues: missing dendritic shafts or spines **(A–E)** and unattached components **(C,F)**. **(A–C)** Display the maximum projection of the confocal images, and **(D–F)** show their corresponding segmented image. Manually segmented spines are displayed in dark blue, whereas dendritic shafts are represented using dark green. **(A,D)** The main apical dendrite (and not the collateral dendrites) was selected to be reconstructed. **(B,E)** Non-segmented collateral spines and dendritic shafts are, especially troublesome when they are close to the main branch. **(C,F)** The annotated image shows gaps in the dendritic shaft and spine necks, mainly because the dendritic shaft and some spines were reconstructed using several surfaces.

**Weak annotations:** Weak annotations are sparse or noisy reconstructions. In our case, small structures (such as spine necks) were not always clearly visible due to the diffraction of the confocal microscopy. Furthermore, neuroscientists sometimes select a region of interest (ROI) and thus do not reconstruct the whole confocal image stack (see Figure 2). We identified two weak annotation problems in our data set (see Figure 4):

- **Missing reconstructions**: some dendrites were not selected to be reconstructed. This problem is, especially relevant when the non-segmented data is close to segmented spines and dendritic shafts (see Figures 4B,E). In order to build the largest GT possible, we did not remove this data. Instead, we tried to alleviate this issue during the training step using several strategies.
- **Unattached components**: occasionally, some parts of dendritic shafts and spines could not be segmented, leaving gaps in between. Unconnected parts were reattached at the preprocessing phase.

In order to increase the quality of our GT, we designed a fully automatic data preprocessing module to solve the problem of unattached components. The objective of this stage was to help the network to learn the topology of dendritic shafts and spines and take into account that they must be connected. We selected the parameters of the following algorithms to provide good overall results. More accurate reconstructions will require intervention from the experts.

**Figure 5:**
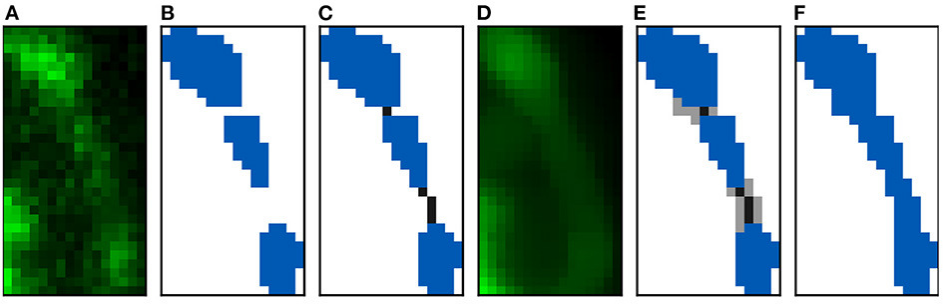
Reconstruction algorithm steps: **(A,B)** Show the clipped maximum projection of a spine composed of three components. **(A)** Shows the confocal image and **(B)** its reconstruction. **(C)** Displays the two paths created to join the three components. **(D)** Shows the confocal image after applying a median filter. The flooding phase results can be found in **(E)**. Finally, **(F)** illustrates the final reconstruction.

The reconstruction process works as follows: first, it is applied to the dendritic shaft, filling in the gaps between detached parts. Then, each spine is processed independently. Finally, spines are connected to the dendritic shaft when needed. These three steps use the same automatic algorithm (see Figure 5). Our algorithm receives the raw confocal image, the segmented image of the dendritic shaft and spines, and the index of the segmented structure to be processed; and it performs the subsequent steps:

1. **Clipping**: this phase computes the axis-aligned bounding box^4^ of the microanatomical structure. Both the segmented and the confocal images are clipped using this bounding box. This step speeds preprocessing up by reducing the volume of data.
2. **Unconnected component detection**: the disconnected parts of the structure are computed using an algorithm to detect all connected groups of voxels (Fiorio and Gustedt, 1996; Wu et al., 2005). The process stops once the structure is composed of a single group of connected voxels.
3. **Path finding**: all the components are traversed consecutively, computing a path to join them to another component. Our path minimizes its length while maximizing the density of the voxels it passes through. We use the A^*^ algorithm (Hart et al., 1968) to minimize the following equation:
  (1) $$\begin{array}{l} \underset{p\in P}{\text{minimize}}\;\sum_{i=1}^{\#p}\left(1-den(p_{i})\right)*f_{s}+\;\\ \;\sum_{i=2}^{\#p}dist(p_{i-1},p_{i}), \end{array}$$
  where a path *p* is an ordered set of adjacent voxels, and *#p* is its cardinality; *P* is the set of all paths that connect the processed disconnected component with another component of the structure and do not intersect other anatomical structures; *p*_*i*_ is the *i*th voxel of *p*; *dist*(*p*_*i*_, *p*_*j*_) computes the center distance between *p*_*i*_ and *p*_*j*_; *den*(*p*_*i*_) returns the normalized density value of *p*_*i*_; and *f*_*s*_ is a scale factor. We found that the path is not too sensitive to changes in the *f*_*s*_ value, and we used *f*_*s*_ = 1.
4. **Noise filtering**: In this step, the algorithm applies a median filter to remove noise from the confocal image. We use an 8 × 8 × 2 voxel mask.
5. **Flooding**: the algorithm uses the path elements (seeds) and the filtered confocal image as inputs for a flood fill algorithm. We set the parameter of the flood fill algorithm to label as spine or shaft (depending on the case) voxels connected to the seeds with intensity values within plus or minus 10% tolerance from the seed intensity. Finally, the algorithm requires the definition of two axis-aligned ellipsoid masks to constrain the minimum and the maximum path growth. We selected a maximum mask size of 3 × 3 × 2 voxels to connect dendritic components and a size of 6 × 6 × 2 voxels for the spine connections. In both cases, the minimum size was 1 × 1 × 0 voxels.

**Figure 6:**
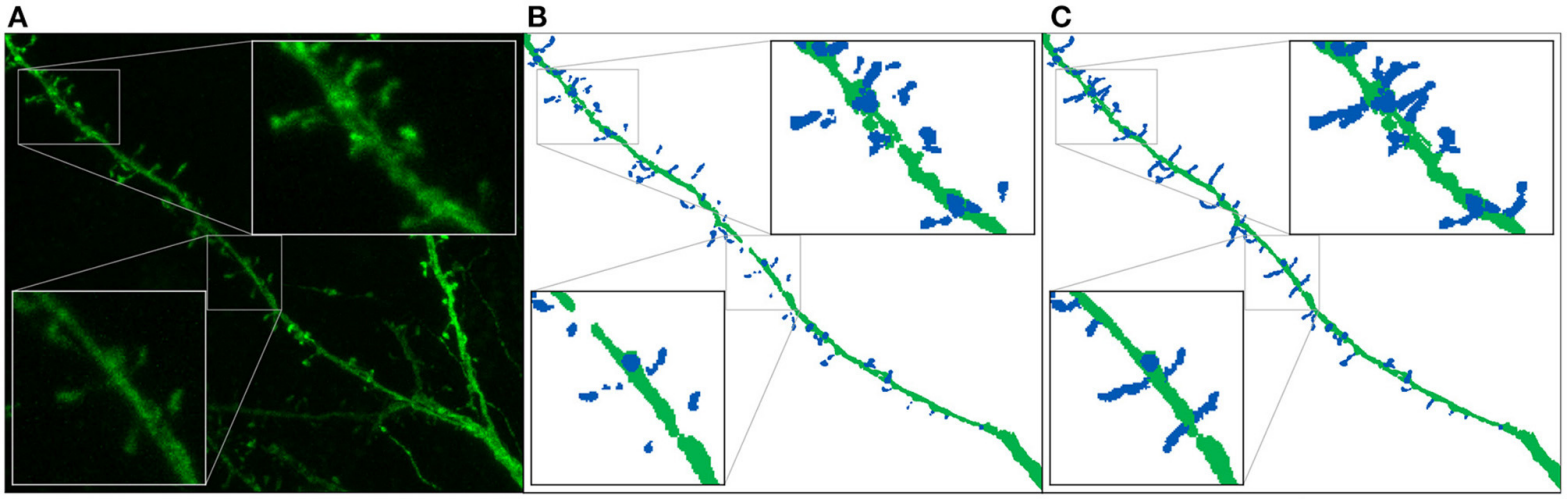
Ground truth preparation: This image shows the maximum projection of a confocal image **(A)** and its corresponding annotated image before **(B)** and after **(C)** the preprocessing. We use dark blue and dark green to represent the manually segmented spines and the dendritic shaft, respectively.

Figure 6 shows an example of a segmented image before and after preprocessing. Before the data augmentation, our GT consists of 62 confocal microscopy images and their corresponding preprocessed segmented images. The confocal images are single 16-bit color channel, whereas the segmented images classify all voxels as background, dendritic shaft or spine.

### 3.3. Network Architecture and Training

In the present study, we took the 3D U-Net (Çiçek et al., 2016) and V-Net (Milletari et al., 2016) architectures as a reference to find the most suitable DL network to solve our problem. This section first describes the most relevant hyperparameters tested to adapt these two architectures. We then provide some details on the training step. Finally, we present the models selected for our segmentation workflow.

#### 3.3.1. Tested Hyperparameters

This section looks at the hyperparameters tested to build the network architecture for segmenting dendritic spines.

***Reference architecture***: our neural networks are based on the two architectures mentioned above. Both models perform convolutions to extract data features, compressing the data first and decompressing it afterwards. Convolutional layers are grouped in stages. 3D U-Net has a fixed number of convolution layers per stage, while V-Net implements a progressive number of layers per stage, from one to three. 3D U-Net downsamples the information using max pooling^5^, and V-Net compresses data by applying 2 × 2 × 2 convolution kernels with stride 2. Both neural networks upsample data through transposed convolutions, but 3D U-Net considers context information beyond the output size. Nevertheless, the most relevant difference between these models is that V-Net was designed to learn a residual function adding the input to the last convolutional layer of the stage. Finally, as in the original study (Milletari et al., 2016), our V-Net implementation uses a PReLU (parametric ReLU) activation function, while 3D U-Net uses a ReLU.

***Loss function***: imbalanced classes are common in image segmentation. U-Net (Ronneberger et al., 2015) tackled this issue proposing a Weighted Cross-Entropy Loss (WCEL) function. Let Ω be the set of all possible pixel positions. Let *p*_*k*_:Ω → [0, 1] be the model prediction for class *k*. Let *r*_*k*_:Ω → {0, 1} be the GT probability map for the class *k*. Since we use one-hot encoding, *r*_*k*_(**x**) takes the value 1 when the pixel **x** is classified as *k* and 0 otherwise. Then, the WCEL is defined as:


(2) $$\text{WCEL}=-\sum_{x\in \Omega }w(x)\sum_{k\in \{0,1,2\}}r_{k}(x)\text{log}(p_{k}(x)),$$

where *w*:Ω → ℝ is a weight map and is computed as *w*(**x**) = *w*_class_(**x**)·*w*_*pixel*_(**x**), where *w*_*class*_:Ω → ℝ deals with imbalanced classes, and *w*_*pixel*_:Ω → ℝ allows the setting of the pixel importance. Let *w*_*k*_∈ℝ be the weight of a class *k*, then:


(3) $$w_{class}(x)=\sum_{k\in 0,1,2}w_{k}r_{k}(x).$$

During our preliminary experiments, we tested different approaches, but we achieved significantly better results with the following smoothed function:


(4) $$\begin{array}{l} \begin{array}{cc} {w_{k}}^{\prime } & =\max\left(\text{log}\left(2\frac{\#\Omega }{\#k}\right),1\right),\\ w_{k} & =\frac{{w_{k}}^{\prime }}{\underset{i\in 0,1,2}{\sum }{w_{i}}^{\prime }}, \end{array} \end{array}$$

where *#Ω* is the number of image voxels and *#k* is the number of image voxels classified as *k*.

**Figure 7:**
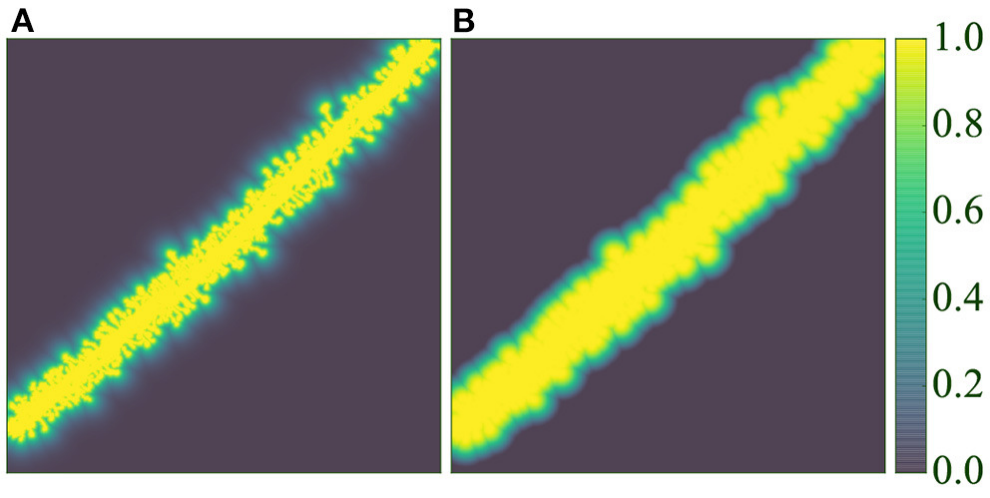
Distance weight maps: **(A)** uses (5) with *r*_*decay*_ = 0.5, and **(B)** uses (6) with *d*_*max*_ = 5 μm.

Section 3.2 explains that some anatomical structures were not segmented by the experts (see Figures 4A,B), hindering the model training. We designed two different weighting functions *w*_*pixel*_ to alleviate this problem. Both functions ($w_{pixel}^{exp}$ and $w_{pixel}^{window}$) are based on the same principle: they reduce the importance of the pixel when the distance to the segmented structures (dendritic shafts and spines) increases. Let *d*:Ω → ℝ be the distance field to the nearest structure, then:


(5) $$w_{pixel}^{exp}(x)={\left(1-r_{decay}\right)}^{d(x)},$$

and


(6) $$\begin{array}{l} w_{pixel}^{window}(x)=\{{\left(1-{\left(\frac{d(x)}{d_{\max}}\right)}^{2}\right)}^{2}\text{ if }d(x)<d_{max}0,\\ \text{ if }d(x)\geq d_{max} \end{array}$$

where the decay ratio (*r*_*decay*_∈[0, 1]) allows control of the exponential decay, and *d*_*max*_ allows the definition of a maximum influence distance in the window function. Figure 7 compares both functions.

It should be noted that the non-weighted version of the cross-entropy loss function was also tested (*w*(**x**) = 1, ∀**x**∈Ω).

Milletari et al. (2016) tailored an objective function for the V-Net based on the Dice coefficient. This function was designed to deal with strong class imbalance cases. Their approach is limited to binary segmentation. Consequently, in our multi-class problem, we used the Generalized Dice Loss (GDL) function (Sudre et al., 2017), which is also based on the Dice coefficient:


(7) $$\text{GDL}=1-2\frac{\sum_{k\in \{0,1,2\}}w_{k}\sum_{x\in \Omega }r_{k}(x)p_{k}(x)}{\sum_{k\in \{0,1,2\}}w_{k}\sum_{x\in \Omega }(r_{k}(x)+p_{k}(x))},$$

where the class weights *w*_*k*_ are computed as follows:


(8) $$w_{k}=\frac{1}{{\left(\sum_{x\in \Omega }r_{k}(x)\right)}^{2}}.$$

***Model size***: we used the same number of layers per stage as described in the original papers. We increased the input and output patch size to observe the impact of augmenting the context information. Moreover, we tried to expand the number of stages (4 and 5 stages) and the initial number of feature channels (8, 16, 32, and 64 channels) which determines the size of all convolutional layers.

#### 3.3.2. Training Step

All convolutional neural network (CNN) models were trained on a preprocessed data set (see Section 3.2). We randomly reserved 10% of the data (6 images) for testing and 10% (6 images) for validation. The remaining data (50 images) were used for training. The images were dynamically divided into patches, depending on the architecture input size. To reduce the problem of non-segmented data, we only fed the network with patches in which dendrites were segmented. Removing unsegmented patches is, especially relevant for unweighted loss functions. The batch size was limited to 8 patches by GPU memory. The training phase was run in a server with two Intel Xeon Gold 6248 CPUs at 2.50GHz, and a NVIDIA Tesla V100 GPU with 32 GB VRAM. We trained our models for a maximum of 50 epochs (around 36 h). As explained in Section 1, overfitting is a frequent problem in ML. The analysis of learning curves is the most commonly used approach to detect this situation. When the loss function decreases in the training set and the error metric increases in the validation set (Section 1), overfitting problems are very likely. In our case, the evolution of the error in the validation set is noisy, but, for most models, the overfitting pattern is distinguishable after 50 epochs. We provide some examples of learning curves as Supplementary Material. For each architecture, we chose the model with the smallest validation error.

#### 3.3.3. Selected Models

During the training phase, we used the validation data to screen the best architectures and tune other learning hyperparameters such as regularization, normalization, dropout, optimizer, and learning rate. To achieve this goal, we computed three quality metrics:


(9) $$\begin{array}{l} Precision=\frac{TP}{TP+FP}, \end{array}$$

(10) $$\begin{array}{l} Recall=\frac{TP}{TP+FN}, \end{array}$$

(11) $$\begin{array}{l} F_{1}\text{-score}=2*\frac{Precision*Recall}{Precision+Recall}. \end{array}$$

In the data set used in this study, only one dendritic branch was selected to be segmented per confocal image (see Section 3.2). Thus, performing an accurate and objective evaluation of the DL models was not a trivial task. To alleviate this problem, we applied a binary mask to remove data from the predictions. Our filter does not consider voxels that are farther away than 7.58 μm from the dendritic shaft, since this was the maximum distance from a segmented spine voxel to the dendritic shaft in our data set. We guided the hyperparameter search using the results from the validation set:

**Reference Architecture:** Although V-Net is known for its ability to learn a residual function, 3D U-Net yielded better results for our particular problem. For similar setups, V-Net performed significantly worse. Therefore, we abandoned it at an early stage.

**Loss function:** Our models had to face weak annotation and imbalanced class problems. In this scenario, WCEL provided better results than unweighted loss functions (even when removing unsegmented patches), GDL, and non-smoothed class weight functions. GDL and non-smoothed class weight functions increased the recall, but the overall model behavior worsened. Regarding WCEL, $w_{pixel}^{exp}$ and $w_{pixel}^{window}$ offered similar results when combined with the smoothed version of *w*_*class*_.

**Data augmentation:** The loss curves' analysis showed that data augmentation alleviates overfitting. For our models, applying simple isometric transformations was sufficient.

**Model size:** Larger patch sizes improved the network accuracy until overfitting could not be handled with data augmentation. Increasing the patch size increases the context information and might improve the model accuracy but requires a larger training set.

**Padding:** Zero padding produced poor results for structures close to the image borders. This problem was alleviated using reflective padding instead.

**Mixed precision:** Finally, since we aimed to run our segmentation on consumer GPUs, we implemented our models using 16-bit floating-point type parameters. We maintained the 32-bit floating-point type for the last layer of each model to ensure numerical stability. This optimization halved our models' memory footprint without any performance loss.

Since the validation set is involved in the training process, the results must be confirmed on unseen data (the test set). It is critical to note that evaluating all models with the test set increases the probability of erroneous inferences. Therefore, we selected a reduced subset of models for evaluation. For reader convenience, we provide the validation results as Supplementary Material.

Based on the performance of the models on the test set, we selected three models: M1, M2, and M3. The results of these three models are shown in Section 4. M1 and M2 exhibit the best *F*_1_-score and balanced precision and recall. Despite using a binary mask, there are spines that the network predicts correctly, which are not segmented in the GT (see Figures 4B,E). Precision is, especially sensitive to wrong false positives, but recall is not. For this reason, we also selected the model with the best recall (M3). M1, M2, and M3 shared most of their hyperparameters: they are base 3D U-Net architecture defined by a 300 × 300 × 66 input patch, a 116 × 116 × 10 output patch, 16 filters in the first layer, and five stages of depth; they use reflective padding and mixed precision; they were trained with augmented data and an Adam optimizer (β_1_ = 0.9, β_2_ = 0.999, ϵ = 10^−7^ and *learningrate* = 10^−7^). Additionally, all of the models use WCEL, but M1 and M2 compute *w*_*k*_ per patch, while M3 uses the whole data set. M1 uses $w_{pixel}^{window}$ (*d*_*max*_= 5 μm), while M2 uses $w_{pixel}^{exp}$ (*r*_*decay*_ = 0.5), and M3 does not use any pixel weight. Instead, during training, M3 only uses patches with more than 10% of the pixels classified as dendrite or spine.

### 3.4. Postprocessing

We developed a GUI application to allow users to segment their confocal stack images using our best DL models (M1, M2, and M3). We then used the algorithm described by Wu et al. (2005) to compute the spine instances. Additionally, users can correct the models' prediction if needed. We designed automatic and user-supervised algorithms to address the following issues: noise, misclassification, unconnected components, and overlapping spines.

***Noise***: given CNN's ability to deal with noisy images, we did not preprocess the inputs. Although the problem is almost negligible, occasionally, our network misclassifies high-intensity background voxels as spines or dendritic shafts. This particular type of misclassification can be solved semi-automatically by eliminating the disconnected dendritic components that verify the following equation:


(12) $$\begin{array}{l} \left({\text{d}}_{V_{d}}\left(x\right)>D\right)\wedge \left(\text{getSize}\left(s\right)<V_{s}\right), \end{array}$$

where d_*V*_*d*__:*S* → ℝ returns the distance from a disconnected component *s*∈*S* to the closest dendritic shaft component bigger than *V*_*d*_, and getSize:*S* → ℝ returns the volume of disconnected component *s*. The user sets the values of *V*_*d*_, *V*_*s*_ and *D*. This filter can be applied to spines, dendrites, or both.

***Misclassification***: this problem is also almost negligible. The users can select a set of pixels and relabel them. We implemented different voxel selection algorithms to facilitate the task. This algorithm can be used to remove the remaining noise if needed.

***Unconnected components***: while detached dendritic shaft sections can be connected automatically, spines cannot. Determining whether or not they must be connected to a dendritic shaft or spine is not a trivial task. For the latter case, we implemented a semi-automatic version of the algorithm described in Section 3.2. Before running the A* algorithm, the user must select which components will be connected. Additionally, they can set the ellipsoid mask sizes, median mask size, and the tolerance of the flood fill algorithm.

***Overlapping spines***: this is the most important issue as it requires a higher degree of user intervention. During the segmentation, some spines appear attached in a connected set of voxels. To address this issue, we allow the users to mark the spine borders manually. In addition to this technique, we implemented a watershed separation algorithm (Beucher and Lantuéj, 1979). The user selects a set of seeds manually [similar to the procedure used in the technique proposed by Das et al. (2021)].

**Figure 8:**
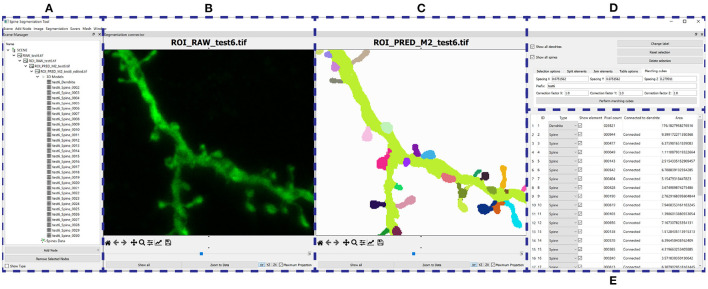
GUI: **(A)** Organizes the users' data hierarchically. **(B–E)** Provide a GUI to the postprocessing algorithms. **(B)** Shows the raw confocal image of the ROI. **(C)** Displays the output segmentation. **(D)** Allows the user to configure the algorithms, and **(E)** shows all the segmented structures.

Our GUI application is shown in Figure 8. The software was implemented in Python, Qt and VTK, following a flexible and modular plugin-based architecture. Due to incomplete segmentation in some GT images, it is impossible to assess the best model quantitatively and objectively. To alleviate this problem and achieve the best segmentations, we make several models available to the user, who can choose the best model for their data. Furthermore, they can segment the whole image or just a user-defined ROI. The tool incorporates basic image editing features and several view modes, and users can choose the ones that best suits their needs. Finally, we implemented the marching cubes algorithm (Lorensen and Cline, 1987) to extract a B-rep from the segmented image. We present case studies of the postprocessing stage in Section 4.3.

## 4. Results

In this section, we examine the results of our models on the test data set. First, we analyze the best models quantitatively (Section 4.1). We then show examples of the strengths and limitations of these models (Section 4.2) and, in the next section (Section 4.3), describe how these limitations can be overcome with our postprocessing stage. Finally, we assess the impact of the preprocessing on the model training (Section 4.4).

### 4.1. Quantitative Analysis

**Figure 9:**
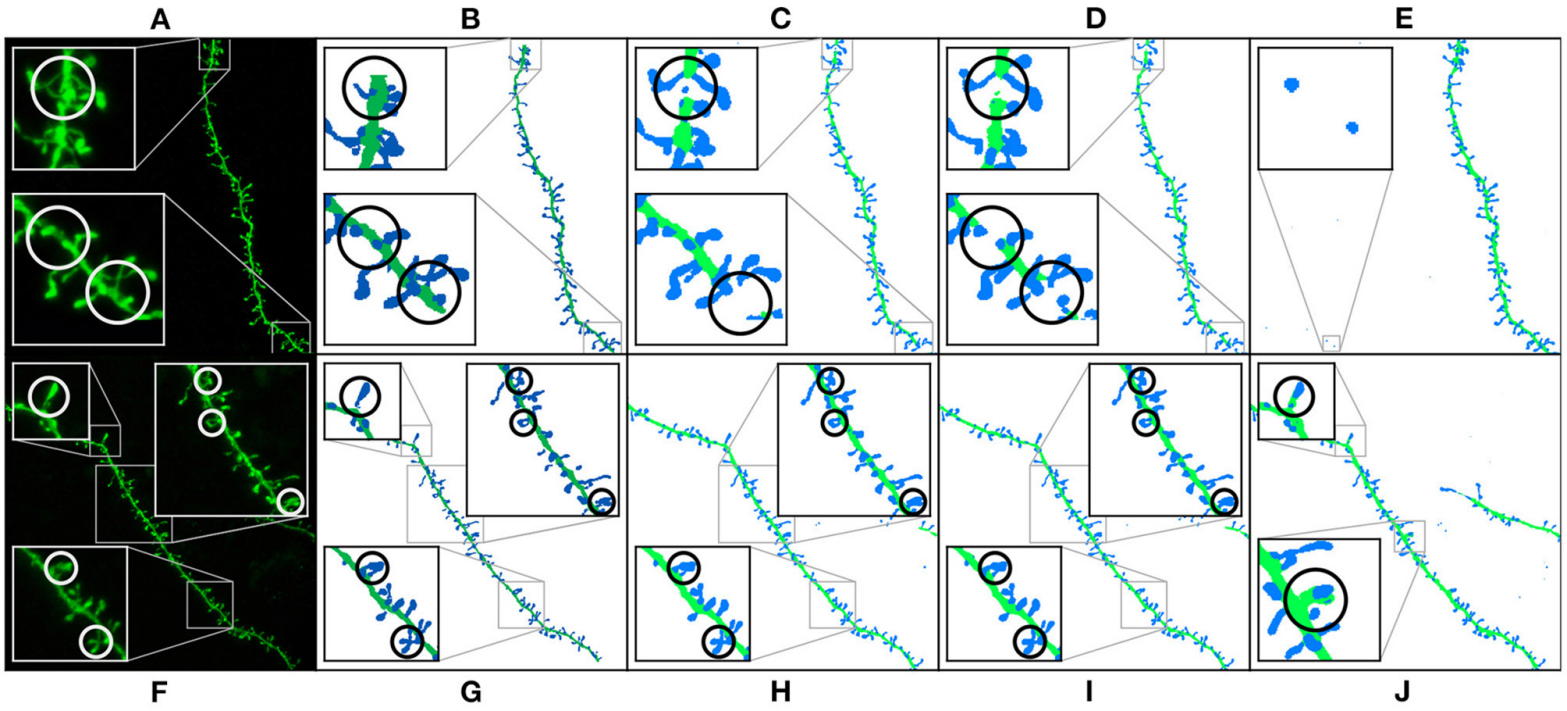
Results of running M1, M2, and M3 models on two images of the test set: This figure shows the maximum projection of two confocal images **(A,F)**, the GT segmented images **(B,G)**, M1 results **(C,H)**, M2 results **(D,I)**, and M3 results **(E,J)**. The following issues are highlighted: unconnected components **(C,D)**, overlapping spines **(H,J)**, noise **(E)**, and misclassification **(J)**. We use dark colors to show the manual segmentation (GT) and bright colors for network predictions. The segmented spines are shown in blue and the dendritic shafts in green.

Table 1 shows the test results of the three selected models. M1 and M2 maximized the *F*_1_-score. Both models balance precision and recall. However, M1 performs slightly better on dendritic shafts and M2 on spines. As mentioned previously, M3 has the best recall. This metric is less sensitive to wrong false positives. However, if the precision value is too low, the misclassification and noise problems increase (see Figure 9). Although noise can be automatically removed in postprocessing, misclassified segmentations require user intervention (see Section 3.4). Nevertheless, since these models can perform well on difficult confocal stacks (see Figure 11), our GUI application allows users to select between the three models.

**Table 1:** Test results of the selected models.

**Model ID**	**Metric**	**Class**		**F_1_-score** **mean**

		**Dendritic shaft**	**Spine**	
M1	*Precision*	74.5 %	66.1 %	77.3 %
	*Recall*	86.9 %	84.9 %	
	*F*_1_-score	80.2 %	74.3 %	
M2	*Precision*	72.5 %	64.9 %	77.3 %
	*Recall*	88.8 %	88.3 %	
	*F*_1_-score	79.8 %	74.8 %	
M3	*Precision*	53.9 %	56.1 %	67.9 %
	*Recall*	90.1 %	87.0 %	
	*F*_1_-score	67.5 %	68.2 %	

### 4.2. Visual Inspection

Figure 9 shows how M1, M2, and M3 perform on two images of the test set. Although all models suffer from the problems described in Section 3.4, most spines are correctly segmented and do not require human intervention. M1 and M2 show problems at the image border since there is less context around the segmented patch. By contrast, M3 can handle challenging scenarios such as image borders, but it is, especially sensitive to noise and exhibits more misclassification cases. Although all models may exhibit these issues, for the sake of clarity, not all subfigures in Figure 9 show all error types.

### 4.3. Case Studies

To incorporate our DL models into the neuroscientists' workflow, we implemented a GUI application that integrates the best networks and the postprocessing algorithms described in Section 3.4. This software also includes several data visualizations and basic editing algorithms. Additionally, it allows the users to extract the data mesh-based B-rep. Our system offers neuroscientists the possibility of segmenting full images on their lab PCs. We tested our system on a PC with an NVIDIA GeForce GTX 1080Ti, obtaining the prediction in 210 seconds for an image size of 1024 × 1024 × 101.

**Figure 10:**
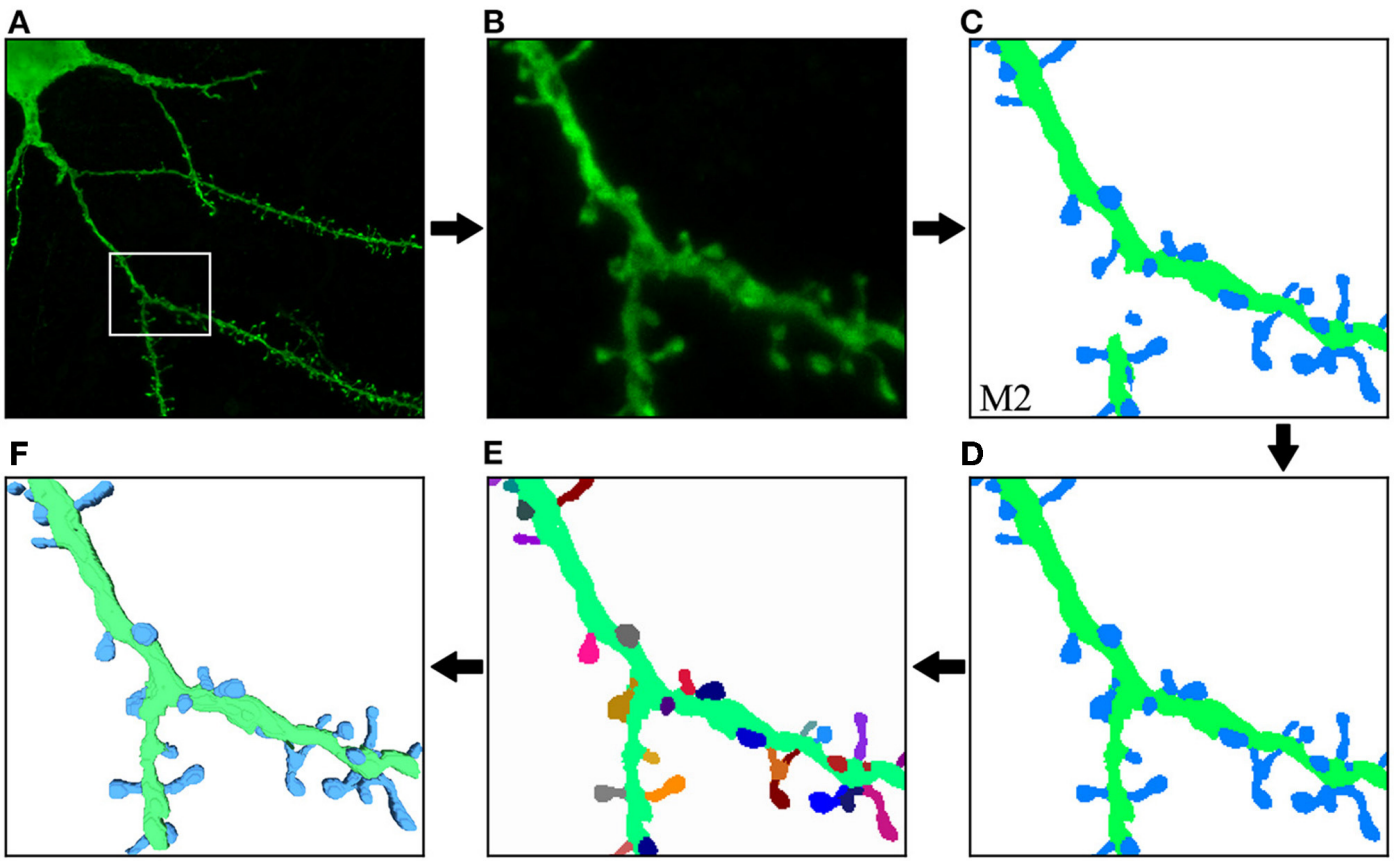
First case study: **(A)** original confocal image; **(B)** ROI selected by the user; **(C)** M2 segmentation; **(D)** correction of misclassified and unconnected components; **(E)** the segmentation after the separation of overlapping spines. Finally, dendritic shaft and spine surfaces are calculated **(F)**. The top row shows the process steps from left to right, and the bottom row continues from right to left. This order allows direct comparison between the preceding and successive images at each step.

Figure 10 illustrates the process and results of a first case study. In this example, the user was not interested in segmenting the entire image. Instead, the neuroanatomist selected an ROI (Figure 10B). M2 was then used to segment the data (Figure 10C). Next, the two unconnected dendritic branches were reattached. The user selected the two components and ran the corresponding algorithm. The width of the union was adjusted by changing the tolerance of the flood fill algorithm. The user then corrected a few misclassified elements, selecting the component and changing its class. Figure 10D shows the segmentation after these two processes. No noise issues needed to be fixed. Once the segmentation was corrected, the user executed a pixel cluster algorithm to estimate single spine instances. At this point, the only problem left was overlapping spines. To address this issue, the user manually selected seeds and ran a watershed algorithm. Solving the issue of overlapping spines requires more user intervention than other postprocessing algorithms. However, our tool requires significantly less user effort than other modern state-of-the-art techniques, such as Das et al. (2021). The results of this step are displayed in Figure 10E. Finally, the dendritic shaft and spine surfaces were extracted (Figure 10F). Figure 8 shows the GUI and the postprocessing panels after computing the B-reps of the segmented structures. A hierarchical scene tree is displayed in panel *A*. This panel allows the operations performed on the data to be tracked. In this example, the user stored the original raw image, the ROI, the segmentation performed by M2, the corrected segmentation, the surface meshes and a tabular structure with dendritic shaft and spine data. Panels *B*, *D*, *F*, and *G* were designed to assist users during the postprocessing.

**Figure 11:**
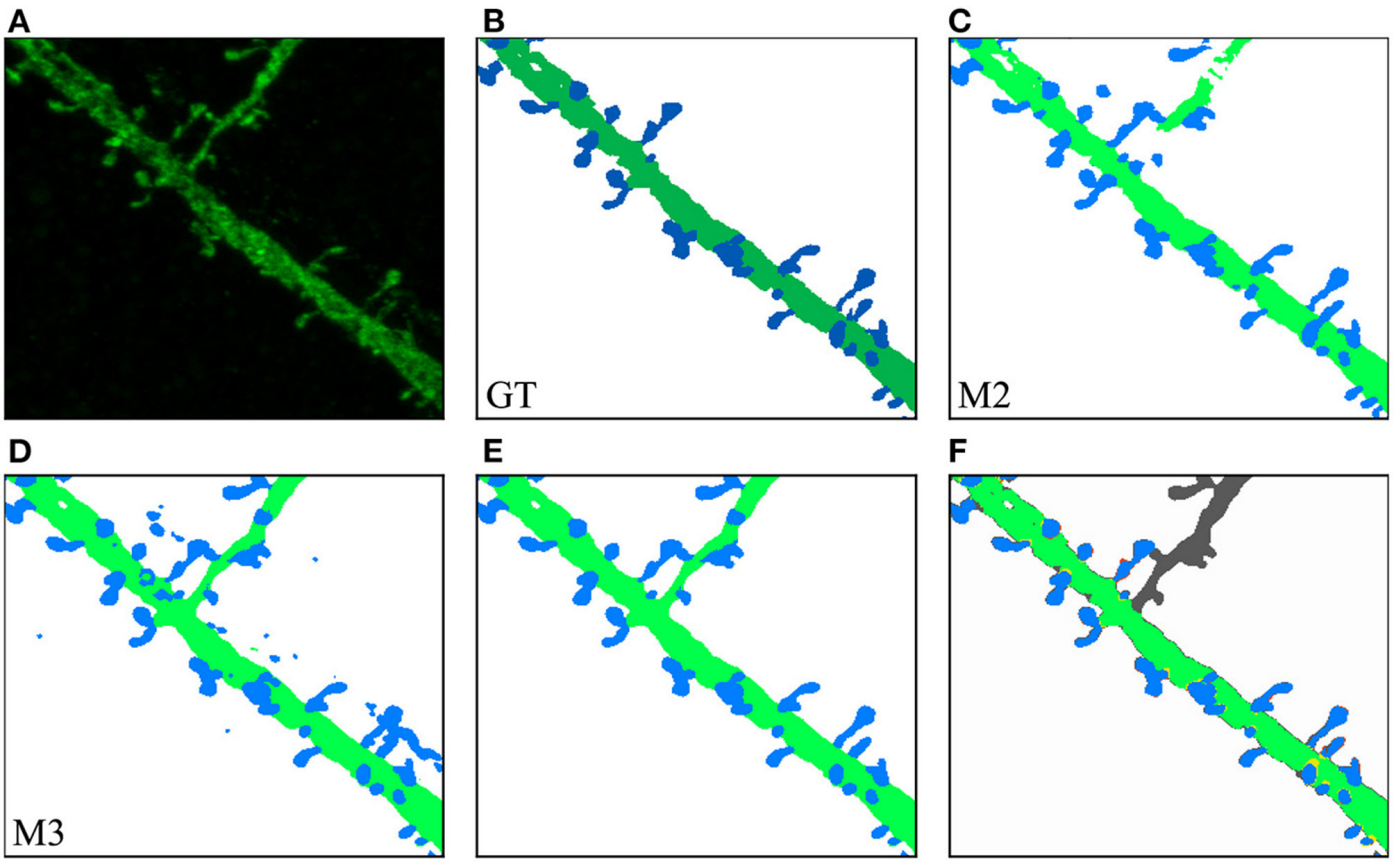
Second case study: **(A)** an ROI selected by the user from the original confocal image; **(B)** GT segmented dendritic shafts and spines (note that the collateral branch was not originally selected for reconstruction); **(C)** M2 produced insufficient quality results segmenting some structures; **(D)** M3 performed better but introduced noise; **(E)** removed noise, the segmented structures in the GT **(B)** were properly segmented; **(F)** compares the GT with M3 results after noise removal, showing matched dendritic shafts (bright green) and spines (bright blue), structures missing in GT (gray), structures missing in M3 (red), and misclassifications (yellow).

In our second case study, the user was interested in a challenging ROI (see Figure 11). In this case, M2 results were not good enough in one of the dendritic branches (Figure 11C). M3 segmented all relevant structures at the cost of increasing the image noise (Figure 11D). The user eliminated noise by removing unconnected spines and dendritic shafts and selecting the following parameters: *D*= 3 μm, *V*_*d*_= 0.16 μm^3, and *V*_*s*_= 0.024 μm^3. These have been set as the default parameters since they work well with our data. Users can modify these values if necessary. There is almost no visual difference between the segmented image after the noise removal process (Figure 11E) and the structures in the segmented images of the GT (Figure 11B).

**Figure 12:**
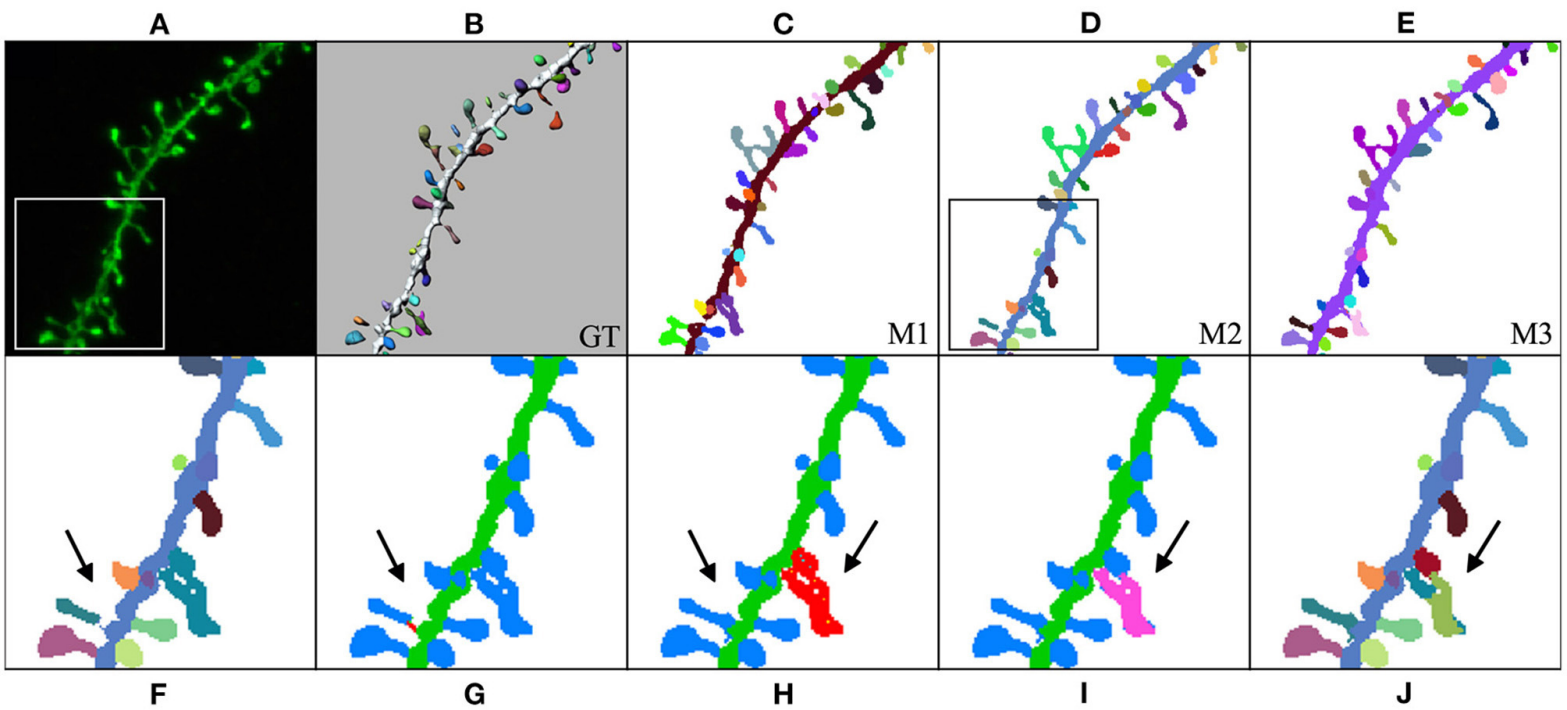
Third case study: **(A)** maximum projection of a confocal image; **(B)** original surface reconstructions used to build the GT; **(C–E)**: M1, M2, and M3 predictions, respectively; **(F–J)** user corrections of M2 predictions. First, the user identified a disconnected spine **(F)**. **(G)** Shows the user's first attempt at attaching the spine to the dendritic shaft. **(H)** Shows the edited final spine reconstruction. Additionally, the user selected three overlapped spines and then separated them in two steps **(I,J)**.

In our third case study (Figure 12), the user computed M1, M2, and M3 predictions and selected M2 to be the model that best fitted the user's expectations. Thereafter, the user could further edit reconstruction errors. For example, in this reconstruction, one of the spines that were not connected to the dendritic shaft could be reattached using our semi-automatic tool. Additionally, spines that were not correctly identified could be separated using our watershed-based algorithm.

### 4.4. Impact of the Preprocessing Stage

The goal of the preprocessing step is to increase the GT quality, detecting disconnected reconstructions of dendritic shafts and spines and reattaching them automatically. M1, M2, and M3 were trained with the preprocessed GT. To assess the impact of the preprocessing step, we trained the architectures used to build M1, M2, and M3 with a non-preprocessed version of the GT and compared their results. We named the DL architectures according to their corresponding models: ArchM1, ArchM2, and ArchM3.

Table 2 compares the total number of unconnected spine parts after segmenting all images of the testing set for the three architectures and the two training sets. As reported previously, the GT does not contain all the structures shown in the confocal image. Therefore, we are only considering the spines predicted by the model that intersect with the spines segmented in the GT. All models trained with a preprocessed GT performed better. However, there was less improvement of ArchM3 than occurred in the other architectures. ArchM3's low precision and high recall make this architecture prone to false positives, oversegmentation, and also more sensitive to noise.

**Table 2:** Comparison of models trained with and without preprocessing the GT using the test set.

**Ratio of unconnected spine parts to total number of spine parts in NPGT test set**	**Arch.** **ID**	**Unconnected spine** **parts in model** **prediction**		**Prediction improvement of the** **model trained on** **PGT**	
		**Trained on** **NPGT**	**Trained on** **PGT**	**With respect to the** **model trained on** **NPGT**	**With respect to the** **NPGT test set**
134 : 451	ArchM1	84	20	76.19%	85.07%
	ArchM2	63	7	88.89%	94.78%
	ArchM3	65	30	53.85%	77.61%

*The third and fourth columns show the number of unconnected spine parts for the models trained with the non-preprocessed GT (NPGT) and the processed GT (PGT). The last two columns present the prediction improvement of the model trained on PGT with respect to the model trained on the NPGT and the NPGT test set*.

**Figure 13:**
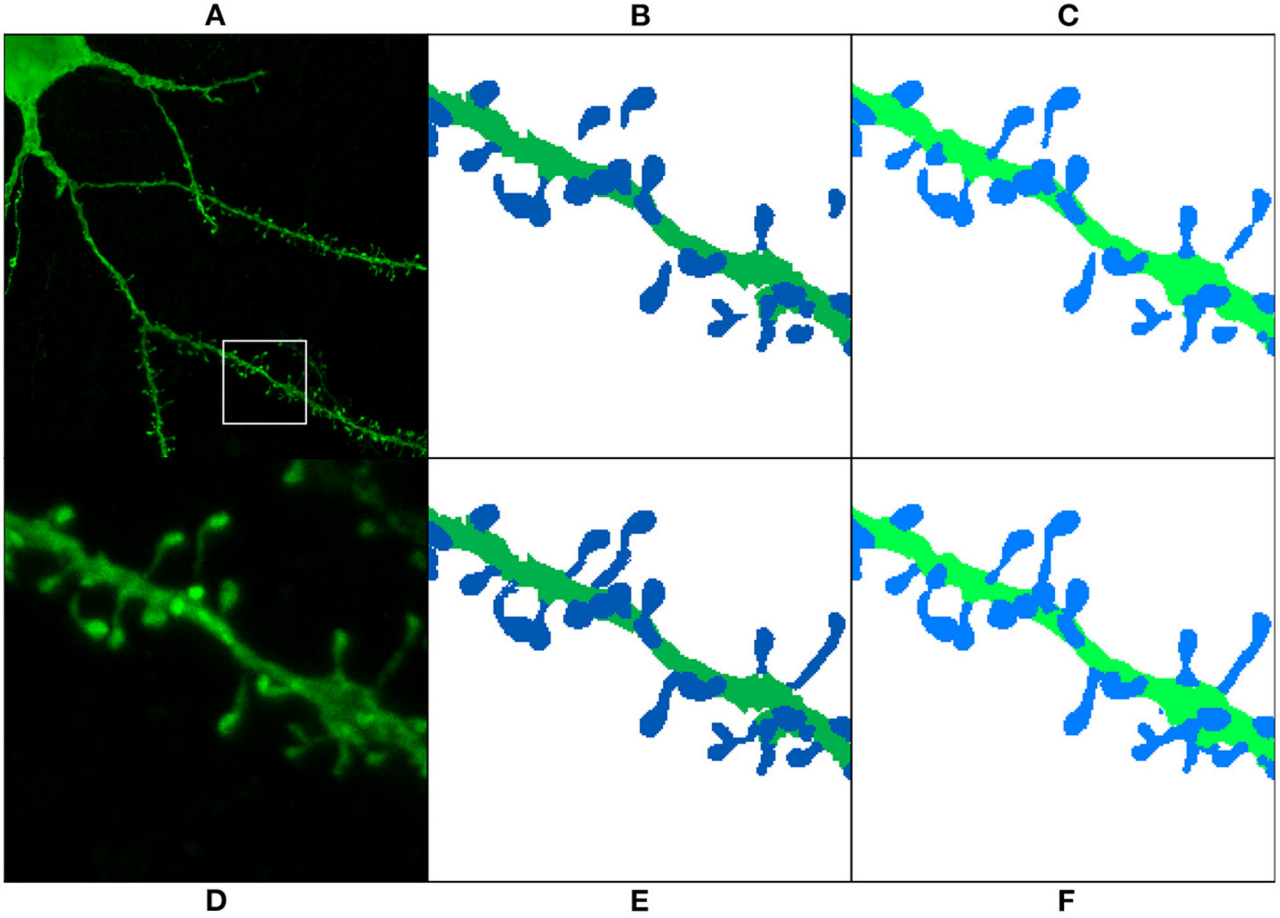
Preprocessing comparison: **(A)** maximum projection of a confocal image; **(D)** ROI segmented in **(B,C,E,F)**. The GT is displayed before preprocessing **(B)** and after preprocessing **(E)**. **(C,F)** Show the prediction obtained with ArchM1. **(C)** Shows the results for the model trained with the non-preprocessed GT, whereas **(F)** displays those of the model trained with the preprocessed GT. We use dark colors to show the manual segmentation (GT) and bright colors for networks predictions. The segmented spines are shown in blue and the dendritic shafts in green.

As mentioned in Section 3.3, we reserved six of the GT images for testing purposes. These images contained the reconstruction of 451 spine components. 134 of these components were disconnected before the preprocessing stage. Table 2 shows that the segmentation generated by all models had a lower number of unconnected spines parts than the manual reconstruction performed with Imaris. Even for the models trained with the non-preprocessed GT, the number of unconnected spine parts decreased. This is not surprising since most spines of the non-preprocessed GT are connected to the dendritic shaft, and the models try to apply this learned behavior to unseen data (the test set). However, a more significant decrease is achieved in the segmentation generated by the models trained with preprocessed GT. Finally, Figure 13 shows the result of ArchM1 trained with non-preprocessed and preprocessed GT. This figure illustrates how M1 connects the spines to the dendritic shaft despite the low intensity of their necks in the confocal image.

## 5. Discussion

The automatic segmentation of dendritic spines using light microscopy continues to present challenges. In the literature, many techniques can be found that resolve certain issues but such techniques also have other limitations. Some techniques focus on providing precise segmentation [such as the one proposed by Das et al. (2021)] but require intensive user intervention. Many others [such as Neurolucida and the technique proposed by Levet et al. (2020)] offer semi-automatic solutions, reducing the time required for the user to complete the task, but hindering the correction of local errors. By contrast, Filament Tracer allows the correction of local errors at the cost of approximating the geometry of the spines and dendritic shafts with simple forms.

Our work demonstrates the possibility of using DL architectures in this context. Artificial neural networks have been successfully applied to segmentation problems in many different domains, providing a fully automatic solution. Despite being popular techniques, they had not previously been used to segment confocal images of dendritic spines. As previously mentioned, the size and quality of the data sets used as GT are essential for training phase success. We managed to build a GT for training reliable DL networks thanks to the quality and size of our non-preprocessed data set. We tackled the remaining problems following three main strategies: data preprocessing, data augmentation, and weighted loss functions. Our preprocessing algorithms joined disconnected structures automatically, increasing the data quality. Section 4.4 shows how our model trained on preprocessed data reconstructs less disconnected parts than Imaris manual reconstructions. The missing structure issues were mitigated by using weighted loss functions and removing unsegmented patches during training. Finally, we significantly reduced overfitting problems using isometric transformations to increase the training data set size. Currently, there are many other data augmentation techniques (Shorten and Khoshgoftaar, 2019). However, exploring them all would go beyond the scope of this research, and we have therefore left their study for future work. We will pay special attention to DL data augmentation techniques such as generative adversarial networks (GANs).

We consider that this work has taken a significant step toward the automation of the segmentation task. However, there is still room for improvement. First, DL networks learn from examples, and our training data set only includes *in vitro* confocal images of healthy humans. Accurately segmenting other image types would require including a sufficient number of examples in the GT. As mentioned in the abstract, obtaining segmented data sets is expensive, and they are rarely publicly available. We provide the source code of all our software implementations. Researchers and laboratories can train our models with their own data sets. Additionally, we provide examples of how our DL models behave on other data types as Supplementary Material. Second, the results obtained show some minor issues. We developed several user-supervised postprocessing algorithms to address these issues and correct local errors, unlike other automatic or semi-automatic systems. Semi-automatic alternatives, such as Neurolucida or the work proposed by Levet et al. (2020), require the modification of the system parameters to refine the initial solution and do not allow the correction of specific errors. However, further segmentation refinement will be necessary to obtain accurate segmented spines in certain cases, particularly regarding neck diameters. Thus, our GUI application integrates models that have demostrated better performance, as well as supervised correction algorithms. The application completely solves the problem of dendritic spine segmentation, although (thus far) this has only been achieved semi-automatically.

Splitting overlapping spines is the most time-consuming correction. This problem may be solved in the future by combining existing spine detection algorithms with the prediction obtained from our DL model. However, we believe that a DL model can solve the problem completely and automatically. To this end, we plan to increase the size and quality of our GT to train new artificial neural networks architectures. Our preprocessing step has proved its effectiveness, allowing us to incorporate corrected segmentations from other tools and our models' outputs, an approach that has been successfully applied to medical imaging in the past (Wang et al., 2018). Such an approach will likely allow us to fully automate the process in the near future.

## Data Availability Statement

The data sets used to build the GT in this study are available on request to the corresponding author RB-P. All code developed for this study can be found in online repositories. The names and links of the repositories can be found in the Supplementary Material.

## Author Contributions

JD, RB-P, and IF-E conceptualized the idea of creating a tool to automatically segment dendritic spines and acquired and segmented the data. IV-G and MG-L designed and implemented preprocessing algorithms, designed and implemented the postprocessing algorithms, and were in charge of its implementation. LT-M, MM-A, and YG-C designed and implemented the first model. IV-G, NC-T, LP, and MG-L designed the subsequent models, which IV-G and NC-T implemented. IV-G, NC-T, and MG-L performed the artificial neural network testing, designed the DL models' test, and wrote the first draft of the manuscript. IV-G, IF-E, RB-P, LP, JD, and MG-L designed the GUI application. IF-E, RB-P, and JD tested the GUI tool. IV-G and NC-T designed all figures. NC-T, IF-E, RB-P, JD, LP, and MG-L contributed to the final version of the manuscript. All authors conceived the project.

## Funding

The research leading to these results has received funding from the following entities: the Spanish Government under grants FPU18/05304, PRE2018-085403, TIN2017-83132-C2-1-R, PID2020-113013RB-C21, BES-2017-081264, TIN2017-85572-P, and DPI2017-86372-C3-3-R and the European Union's Horizon 2020 Framework under the Specific Grant Agreement No. 945539 (HBP SGA3).

## Conflict of Interest

The authors declare that the research was conducted in the absence of any commercial or financial relationships that could be construed as a potential conflict of interest.

## Publisher's Note

All claims expressed in this article are solely those of the authors and do not necessarily represent those of their affiliated organizations, or those of the publisher, the editors and the reviewers. Any product that may be evaluated in this article, or claim that may be made by its manufacturer, is not guaranteed or endorsed by the publisher.

## Footnotes

^1^We will use the term “DL model” to refer to a trained deep artificial neural network architecture. We will use the term “architecture” to refer to the topology of a DL network and the rest of its hyperparameters.

^2^“Segmented images” are images in which their voxels are classified as background, spine, or dendritic shaft.

^3^Overfitting is a common problem in ML. It occurs when the model learns to resolve the task on the training set but does not work accurately on unseen data.

^4^The minimum cuboid (box) whose faces are perpendicular to one of the basis vectors (axis) and encloses a given 3D shape.

^5^3D U-Net max pooling: our implementation applies a pool of 2 × 2 × 2 voxels with stride of 2 × 2 × 1 voxels in the first two stages and a stride of 2 × 2 × 2 voxels in all other stages.
