# Supplementary material for: A Deep Learning-Based Workflow for Dendritic Spine Segmentation
Source: Front Neuroanat. 2022 Mar 17;16:817903. doi: 10.3389/fnana.2022.817903 (PMC8967951; doi:10.3389/fnana.2022.817903)
Supplement: Supplementary file 2 [file Presentation_1.pdf]

Supplementary Material

In addition to the manuscript, we provide the following additional material:

SUPPLEMENTARY VIDEOS

For the readers' convenience, we created the following videos:

- Video summarizing the main features of our GUI application (attached to this document).
- Three video tutorials showing the GUI application functionalities.
  [Link]

CODE REPOSITORIES AND BINARY FILES

To assure the reproducibility of our results, we release all the code developed for this study. This code is distributed under a Dual License model, depending on its usage. For its non-commercial use, it is released under an open-source license (GPLv3). Please contact Marcos García-Lorenzo if you are interested in a commercial license. Additionally, we provide the compiled version of our GUI application for Windows 10. The code and binaries can be found in the following repositories:

- **DeepSpinePreprocessing**. GT preparation algorithms repository.
  [Link]
- **DeepSpineTool**. Code repository of our GUI application for dendritic spine segmentation, also including an executable package compiled for Windows 10.
  [Link]
- **DeepSpineNet**. Code repository of our deep learning framework for dendritic spine segmentation.
  [Link]

MANUALLY SEGMENTED DATA SET

The data sets used to build the GT in this study are available on request to the corresponding author, Ruth Benavides-Piccione.

RESULTS ON VALIDATION AND TEST

Section 4 analyzed the results of the selected models. Attached to this document, we provide a table with the assessment of all models tested on the validation set. This table includes the hyperparameters used in each model. We identify each model with a numerical identifier (ID). M1, M2, and M3 correspond to IDs 132, 145, and 109, respectively.

ADDITIONAL RESULTS

As described in Section 3.1, we trained our models on confocal image stacks from human pyramidal neurons injected with Lucifer Yellow (brain tissue fixed by immersion in paraformaldehyde). To analyze the generalization power of our models to other image modalities and conditions, we tested our models' behavior on two stacks of confocal images from mouse pyramidal neurons (brain tissue fixed by intracardial perfusion of paraformaldehyde). Figure S1 illustrates these results. Although the model has been trained only using human brain images, it produces acceptable results when using mouse brain images. The prediction has a higher noise in the background of the image in comparison with the human data (Figure S1E

and Figure S1F). This is probably due to the fact that dendritic spines are larger and longer in humans compared to mice (Benavides-Piccione et al., 2002), making it easier to segment the human confocal images. Nonetheless, this noise is easily removable through our GUI application (Figure S1G and Figure S1H).

LEARNING CURVES

This section shows the loss curves' analysis during training. We compare the loss function values for the training and validation data sets. Figure S2 clearly shows how the loss function value for the training set always improves, while the results for the validation set eventually get worse. Models 132, 137, and 145 are trained with data augmentation. Their corresponding plots show that overfitting issues occur later, and the slope of the curve is less steep (Figure S2D, Figure S2E, and Figure S2F).

REFERENCES

Benavides-Piccione, R., Ballesteros-Yáñez, I., DeFelipe, J., and Yuste, R. (2002). Cortical area and species differences in dendritic spine morphology. *Journal of Neurocytology* 31, 337–346. doi:10.1023/A:1024134312173

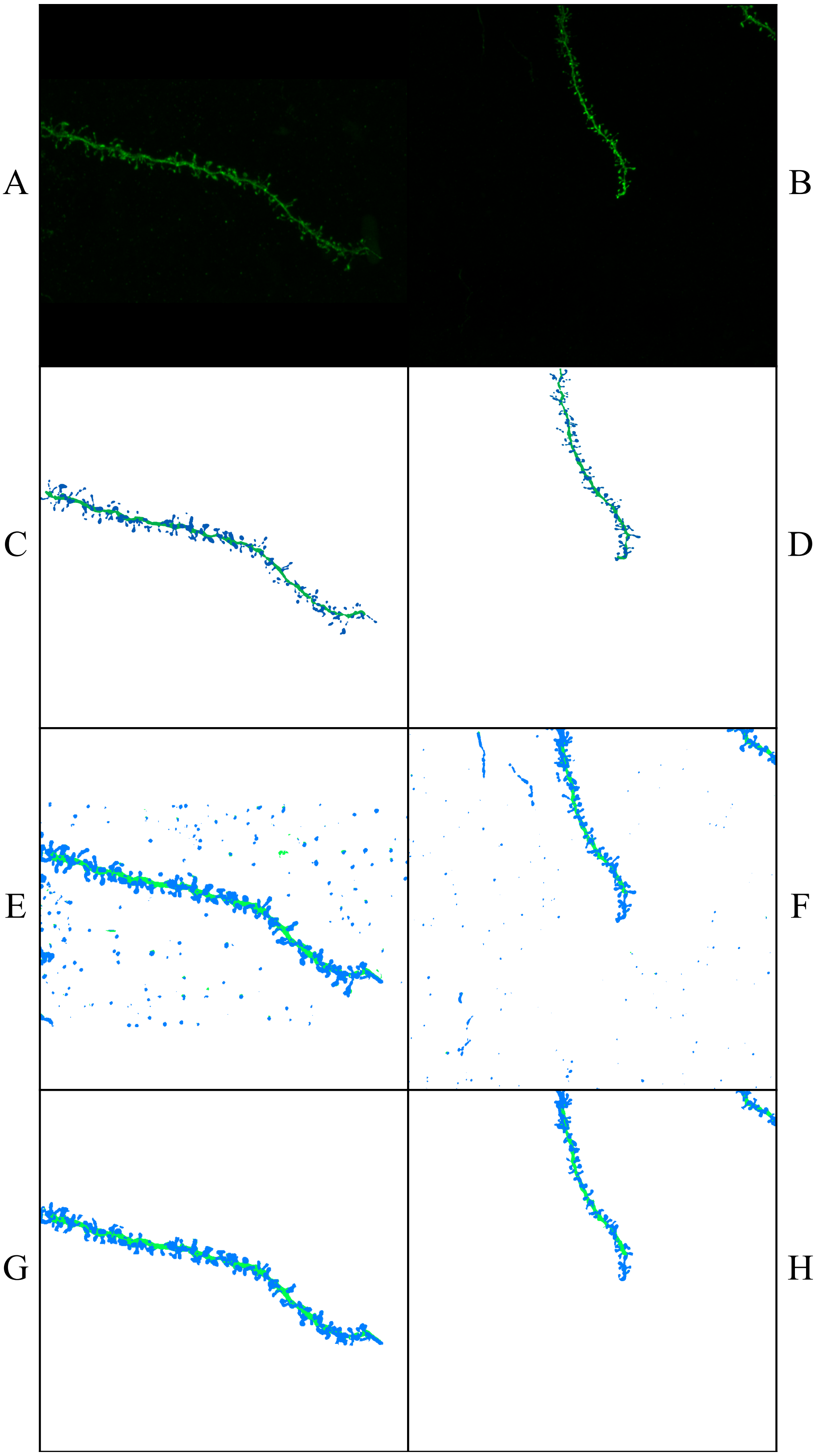

Figure S1. M1 prediction of two mice images. This figure shows the maximum projection of two confocal images ((**A**) and (**B**)), the non-preprocessed manually segmented images ((**C**) and (**D**)), the M1 prediction ((**E**) and (**F**)), and the previous prediction removing the noise removal with our GUI application ((**G**) and (**H**)).

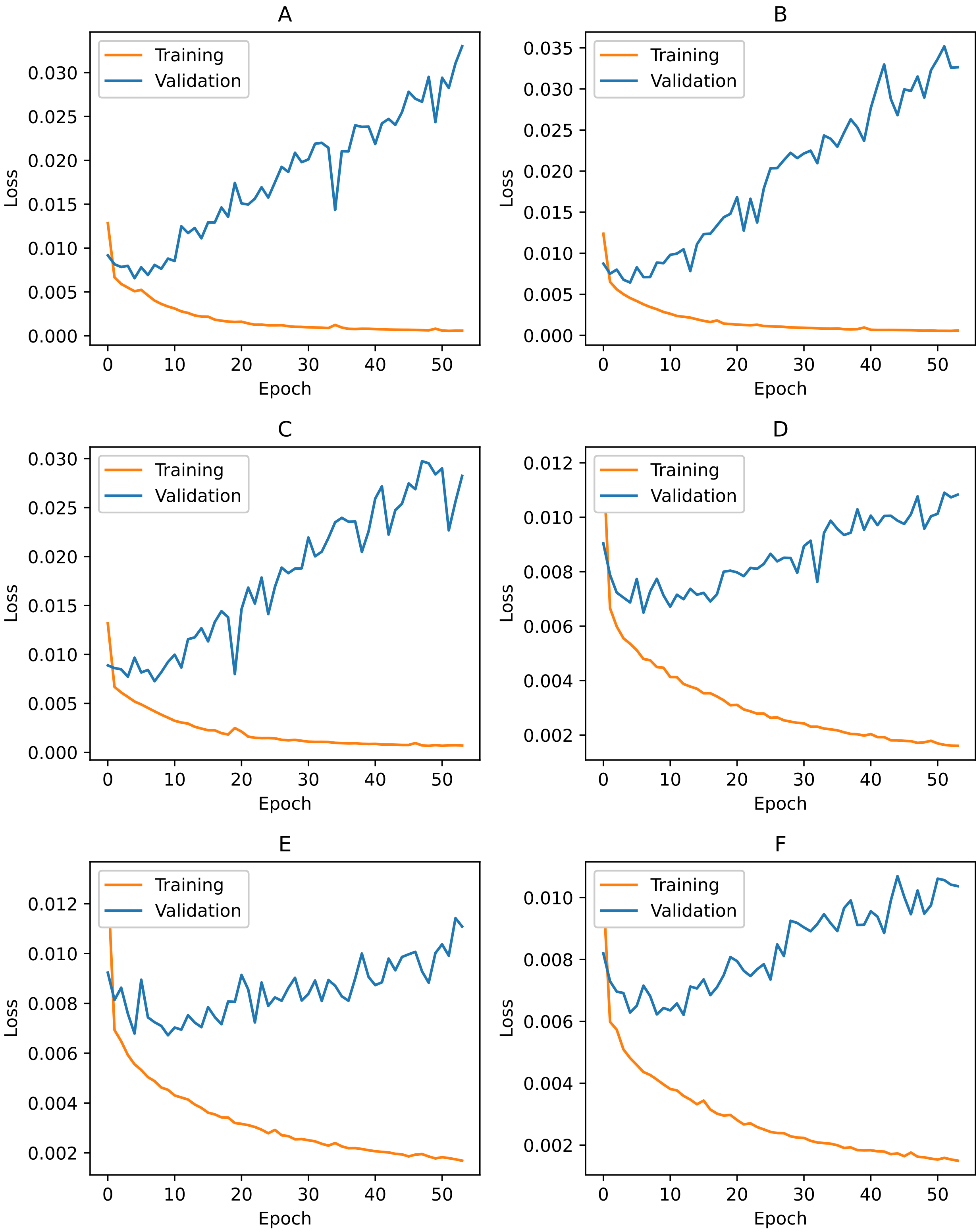

Figure S2. Learning curves. These plots compare the evolution of loss function values for the training and validation sets during the training step. (**A**), (**B**), and (**C**) show the result of models 95, 98, and 111, respectively. These models have been trained *without* data augmentation. (**D**), (**E**), and (**F**) display the evolution of the loss function of models 132, 137, and 145, respectively, which have all been trained *with* data augmentation.
